# Investigating the Impact of Organizational Justice on the Relationship Between Organizational Learning and Organizational Silence in Clinical Nurses: A Structural Equation Modeling Approach

**DOI:** 10.1155/2024/7267388

**Published:** 2024-10-23

**Authors:** Reza Nemati-Vakilabad, Pouya Mostafazadeh, Alireza Mirzaei

**Affiliations:** ^1^Students Research Committee, School of Nursing and Midwifery, Ardabil University of Medical Sciences, Ardabil, Iran; ^2^Department of Medical-Surgical Nursing, School of Nursing and Midwifery, Ardabil University of Medical Sciences, Ardabil, Iran; ^3^McS of Emergency Nursing, Department of Emergency Nursing, School of Nursing and Midwifery, Ardabil University of Medical Sciences, Ardabil, Iran

**Keywords:** clinical nurses, Iran, organizational justice, organizational learning, organizational silence

## Abstract

**Background:** When nurses feel that the learning processes in their organization are fair and just, they are more likely to feel confident about sharing their knowledge, expressing their concerns, and contributing to the learning process. Conversely, suppose employees perceive a lack of organizational justice. In that case, they may be less likely to speak up and share their valuable input due to concerns about unfair treatment or possible negative consequences.

**Objective:** Nurses' silence and organizational learning may have a connection yet to be thoroughly investigated. We are exploring whether organizational justice mediates this relationship by improving nurses' perception of it and reducing silence among them.

**Methods:** A study was conducted in Ardabil, Iran, to analyze the correlation between organizational learning, organizational justice, and organizational silence among 319 healthcare professionals from five hospitals. The study utilized three assessment tools: the organizational learning questionnaire, the organizational justice scale, and the organizational silence scale. The collected data were analyzed using IBM SPSS Statistics, and a structural equation model (SEM) was developed using the bootstrap method in AMOS 24.0 to test the proposed model.

**Results:** Our study found a strong positive relationship between organizational learning and organizational justice and a significant negative correlation between organizational learning and silence. Also, there was a significant negative relationship between organizational justice and silence. SEM showed that organizational learning indirectly affects organizational silence through organizational justice as a mediator, explaining 72.3% of all variance in organizational silence.

**Conclusion:** Our findings indicated that organizational learning is positively associated with justice but negatively associated with silence. When nurses experience organizational justice, they are less likely to remain silent. Encouraging nurses to share their opinions and concerns reduces silence and improves working conditions, morale, and patient care. Further research is needed to understand the complex interplay between organizational learning, justice, and silence in nursing settings.

## 1. Introduction

In today's rapidly evolving healthcare landscape, organizations must prioritize innovation and improvement to enhance patient safety and quality of care. Nurses are crucial in driving change and fostering innovation, directly impacting healthcare outcomes [1]. Their contributions are invaluable and warrant our utmost respect and appreciation. However, healthcare organizations face significant challenges in delivering safe, high-quality care amid constant change and uncertainty [2]. In this context, experts in healthcare management advocate for organizational learning as a promising strategy to address these challenges and improve safety and quality, offering a beacon of hope in the face of these difficulties [3]. Organizational learning involves creating, acquiring, and integrating knowledge to develop resources and competencies that enhance organizational performance [4]. It encompasses implementing strategies and executive plans to improve services through knowledge and better understanding, empowering employees to continually utilize their abilities to identify and comprehend complexities, clarify goals, and develop [5].

Understanding the cognitive and social aspects of learning is crucial for effective knowledge-sharing and organizational changes in healthcare. Integrating learning into daily operations at all levels is vital for organizational success [6]. Continuous learning is essential for nurses' professional development and is a source of motivation. Participation in ongoing learning initiatives helps nurses adapt to rapid changes in knowledge, nursing practices, and healthcare delivery [5, 6]. In a dynamic healthcare environment, healthcare workers must be well-prepared and empowered to voice their opinions, enabling adaptation and continuous improvement [7]. Learning from healthcare workers is beneficial and essential for organizations striving to outperform competitors and enhance safety and quality [8]. The current educational system in universities imparts students with a blend of knowledge and concepts. However, it should also equip them with the skills to analyze, prioritize, and organize new knowledge, necessitating critical thinking and practical learning. Therefore, it is crucial to focus on the organizational learning management of nurses [5].

A significant barrier to effective organizational performance is “organizational silence,” defined as employees' conscious withholding of concerns and ideas about organizational issues [9]. Historically, silence was often equated with loyalty; however, recent studies reveal that a culture of silence can lead to detrimental outcomes within organizations, such as poor decision-making, reduced adaptability, and compromised patient safety and care quality [8]. Employees' reluctance to share honest feedback can hinder decision-making processes, limit organizational adaptability, and reduce the capacity for change, ultimately jeopardizing patient safety and care quality [10]. The inability to address negative feedback can stifle organizational development, reduce efficiency, and foster employee dissatisfaction, leading to stress and deviant behaviors [9].

Furthermore, research indicates that organizational justice, which refers to the fairness and ethical treatment of individuals within an organization, is crucial in shaping employee attitudes and behaviors [8]. It significantly influences the reduction of organizational silence and helps explain employee reactions to perceived unfairness in processes and outcomes [11]. Fair treatment fosters a positive work environment, encouraging employees to speak up, while perceptions of injustice lead to silence [12]. The social exchange theory suggests that a fair organizational climate can mitigate silence, highlighting a negative correlation between organizational justice and employee silence [13].

The Iranian culture's emphasis on collectivism and respect for hierarchy significantly influences nurses' approach to learning in healthcare settings. Prioritizing family and community can create a collaborative environment that values knowledge sharing. However, cultural norms may lead to organizational silence if individuals hesitate to speak up. Understanding these dynamics is crucial for developing strategies that promote open communication and encourage organizational learning among Iranian nurses, ultimately improving patient care and safety. Despite the significance of these dynamics, further research is needed to understand the relationship between organizational learning and silence among nurses [12]. Specifically, it is essential to determine whether nurses' perceptions of organizational justice improve with organizational learning, leading to a reduction in silence. This study aims to address this gap by investigating the role of organizational justice in mediating the relationship between organizational learning and silence among nurses. By elucidating these connections, this research provides valuable insights into how healthcare organizations can foster a culture of transparency and continuous improvement, ultimately benefiting patient care and organizational effectiveness.

## 2. Literature Overview

### 2.1. Organizational Learning and Nurses' Organizational Silence

Experts in healthcare management advocate for organizational learning as a vital strategy for enhancing safety and quality within healthcare settings [9]. Organizational learning is strategically positioned in modern management to enhance employees' skills and foster the organization's adaptability. The organization can effectively respond to market changes and maintain its competitive position by cultivating a dynamic learning environment [6]. Organizational learning, which is a process that fosters positive change in an organization's knowledge, cognition, and collective actions, ultimately improving its capacity to achieve desired outcomes [4], is particularly important in the face of rapid changes and unpredictability in the healthcare environment. It is crucial for healthcare workers to feel empowered to express their opinions and suggestions, as this open dialog is essential for adaptation and continuous improvement [14].

Healthcare workers who voice their concerns are critical in identifying and preventing avoidable patient harm and enhancing patient safety [15]. Organizational silence, which is the conscious choice of employees not to share their concerns and ideas, poses a significant risk in healthcare settings [9]. This silence often stems from fear or distrust, leading to withholding crucial information. This lack of communication can result in poor decision-making, jeopardizing patient safety and care quality. Therefore, fostering an open communication and transparency environment is imperative for mitigating the risks associated with organizational silence [8].

### 2.2. Organizational Justice and Nurses' Organizational Silence

Organizational justice pertains to perceptions of fairness within the workplace and encompasses various dimensions, including distributive justice (fairness of outcomes) and interactional justice (IJ) (fairness of interpersonal interactions) [16, 17]. Research has demonstrated a strong correlation between perceptions of organizational justice and positive workplace outcomes, such as enhanced job performance, increased organizational commitment, and reduced turnover intentions [18]. Furthermore, distributive and IJ significantly contribute to employees' psychological well-being, fostering satisfaction and self-actualization [17]. In nursing, organizational justice is a potent predictor of attitudes and behaviors. Studies have revealed that diminished perceptions of organizational justice are linked with increased organizational silence among nurses [1]. This relationship underscores the importance of fair treatment in promoting a culture where nurses feel valued and respected, and thus, feel comfortable voicing their concerns. This, in turn, enhances patient safety and organizational effectiveness.

### 2.3. Nurses' Organizational Learning and Organizational Justice

In today's fiercely competitive healthcare landscape, the ability of organizations to learn faster than their rivals is a crucial survival factor [19]. Continuous learning enables employees to adapt to ongoing changes and enhances long-term performance by leveraging their skills and values [20]. However, the ramifications of ineffective organizational learning, such as stagnation, poor performance, and declining quality, emphasize the importance of continuous learning. Therefore, it is essential to emphasize this aspect to make healthcare professionals feel more prepared and adaptable. Fostering fair and ethical behavior is crucial to establishing a strong foundation for this learning, as it significantly improves decision-making and creates an environment conducive to learning [21].

Organizational justice is pivotal in this context, as it directly influences how employees perceive their workplace and its learning culture [22]. By promoting an atmosphere of fairness, organizations can enhance perceptions of justice among employees, thereby creating a culture that values shared experiences and knowledge [23]. As leaders, it is our responsibility to ensure that our organizations are fair and just, fostering an environment where everyone feels valued and respected. The dimensions of organizational justice, procedural justice (PJ) and IJ, are particularly relevant, as they can significantly impact employees' willingness to engage in learning behaviors. This culture of equity encourages individuals to learn from their experiences and promotes open communication, reducing the likelihood of organizational silence [24]. Further research is necessary to explore the intricate relationship between organizational learning and organizational justice, mainly how the dimensions of justice influence learning processes. Incorporating current literature on this topic will also strengthen the foundation of this discussion, ensuring a comprehensive understanding of these critical dynamics.

### 2.4. The Mediating Role of Organizational Justice

Organizational learning encompasses the processes organizations acquire, create, and transfer knowledge essential for adaptation and survival in a competitive landscape [25]. Conversely, organizational justice refers to the perceived fairness in workplace interactions, including distributive, PJ, and IJ [26]. Organizational silence, defined as withholding critical information and ideas, can severely impede organizational effectiveness [27].

Existing research on organizational learning, organizational justice, and organizational silence in healthcare has identified notable gaps. This study addresses these gaps by exploring the factors contributing to nurses' fear or distrust, which can lead to silence. While the impact of organizational justice on various outcomes is acknowledged, further investigation is needed into its effects on nursing practices and patient safety, particularly regarding how different dimensions of justice influence nurses' willingness to voice concerns. Our proposed mediation model suggests that employees are more likely to share knowledge and raise concerns when they perceive organizational learning processes as fair. Conducting additional studies will be essential in validating this model and providing insights into the interplay between organizational learning, justice, and silence in healthcare contexts.

#### 2.4.1. Research Hypotheses

**Figure 1:**
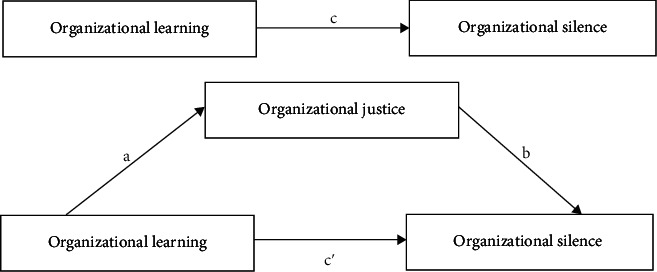
The hypothesized theoretical model.

This study examines the complex interconnections among organizational learning, organizational justice, and organizational silence within nursing. Based on the above theories and literature, we hypothesize the following (Figure 1).

**Hypothesis 1 .**

Organizational learning is correlated with organizational silence.

Rationale: Learning organizations create an environment that encourages the expression of opinions and ideas, which can lead to a reduction in organizational silence. Research has shown that in learning environments, employees are more likely to share concerns and suggestions, ultimately improving care quality and patient safety [8].

**Hypothesis 2 .**

Organizational justice is correlated with organizational silence.

Rationale: Perceptions of injustice in the workplace can lead to employee silence, as individuals may refrain from expressing their views. Studies indicate that distributive and IJ can significantly influence employees' willingness to voice concerns, suggesting that organizational justice can help mitigate silence [12].

**Hypothesis 3 .**

Organizational learning is correlated with organizational justice.

Rationale: Organizations that prioritize learning and employee development tend to foster a fair and transparent environment. This atmosphere makes employees feel valued and motivates them to engage in learning processes. Research indicates that learning organizations often experience higher levels of organizational justice [22].

**Hypothesis 4 .**

Organizational justice mediates the relationship between organizational learning and organizational silence.

Rationale: When employees perceive learning processes as fair and just, they are more likely to feel empowered to share their knowledge and raise concerns. This sense of justice can help reduce organizational silence while promoting organizational learning. Studies suggest that organizational justice can serve as a mediator in the complex relationships between learning and voicing behaviors [13].

Significant papers addressing the topics of the documents are shown in Table 1).

## 3. Methods

### 3.1. Design

We conducted a cross-sectional study on Iranian nurses, adhering to STROBE guidelines for observational studies [28].

### 3.2. Setting and Participants

Data collection for this study was conducted through convenience sampling from December 2023 to March 2024. We selected clinical nurses from five educational-therapeutic hospitals associated with Ardabil University of Medical Sciences in Iran. The choice of these specific hospitals was based on their diverse patient populations and varying clinical settings, which allowed for a comprehensive understanding of the nursing practices across different environments. To be eligible for participation, nurses had to meet specific criteria: they needed at least 6 months of work experience, a willingness to participate, and no history of psychological or emotional disorders, such as depression or anxiety disorder. This criterion was established to ensure that participants could provide reliable responses without the influence of psychological distress.

Nurses who withdrew from the study or did not complete the questionnaire were excluded from the analysis. The sample size was determined based on the guidelines provided by Wolf et al. [29], which recommends a ratio of 5–10 samples per variable (number of items) for structural equation modeling (SEM). Given that our study included 80 items, a sample size between 400 and 800 was deemed desirable, accounting for a 10% nonresponse rate. However, this study's sample attrition rate was 27.5%, resulting in data from only 319 participants being analyzed. Given the initial target range, this sample size provided a robust basis for the findings.

### 3.3. Data Collection

The researchers collected data from a demographic information form, the organizational learning instrument-development stages (OLI-DS) instrument, the organizational justice questionnaire, and the organizational silence behavior scale. The questionnaires were designed using web-based software and sent to clinical nurses through email and virtual messengers (e.g., WhatsApp and Telegram). The participants then completed the questionnaires through a self-administered form.

#### 3.3.1. Demographic Information Form

This form was designed to collect demographic information based on relevant literature. It comprises 6 closed-ended questions, including age, working experience, gender, marital status, education level, and working department.

#### 3.3.2. OLI-DS Instrument

Lyman and Ethington designed the OLI-DS instrument for clinical nurses [4]. The OLI-DS consists of 35 items and four subscales: identity and ownership (IO) (13 items), team and respect (TR) (6 items), accountability and support (AcS) (10 items), and reliability and sustainability (RS) (6 items). The items are rated on a four-point Likert scale (1 = *strongly disagree* to 4 = *strongly agree*). The instrument's score is determined by calculating the mean score for each subscale. Higher scores indicate more significant organizational learning.

#### 3.3.3. Organizational Justice Questionnaire

The organizational justice scale used in the study was developed by the authors in [30]. This scale includes 13 items with two subscales: PJ (7 items) and IJ (6 items). Participants rated their organizational justice using a five-point Likert scale from 1 (*strongly disagree*) to 5 (*strongly agree*). The score range was from 13 to 65. The instrument's score is determined by calculating the mean score for each subscale. Higher scores indicate greater organizational justice. In this study, Cronbach's alpha of the total scale was reported to be 0.96, and its subscales were 0.77 and 0.91.

#### 3.3.4. Organizational Silence Behavior Scale

The organizational silence behavior scale used in the study was developed by Yalçın and Baykal [10]. This scale includes 32 items and has four subscales: silence climate (SC) (5 items), silence based on fear (SBF) (12 items), acquiesce silence (AS) (10 items), and silence based on protecting the organization (SBPO) (5 items). The scale was designed as a five-point Likert scale containing five response options, from 1 (*never stay silent*) to 5 (*always stay silent*). The instrument's score is determined by calculating the mean score for each subscale. As the total score on the scale increases, the level of silence also increases. The scale is self-reported to evaluate participants' behavior towardorganizational silence within their organizations. Cronbach's alpha for the scale was 0.93, and for the subscales, it was as follows: SC: *α* = 0.91, SBF:  = 0.91, AS: *α* = 0.93, and SBPO: *α* = 0.85. The present study confirmed the scale's internal consistency by calculating Cronbach's alpha of 0.91, ranging from 0.83 to 0.89 for the three subscales.

### 3.4. Statistical Analysis

The study utilized IBM SPSS Statistics for Windows, Version 26.0 (IBM Corp., Armonk, New York, USA) to perform descriptive statistics, including frequency, percentage, mean, and standard deviation. These descriptive measures provided a foundational understanding of the participants' demographic characteristics and the distribution of key variables. Pearson correlation analysis was employed to explore the relationships between organizational learning, organizational justice, and organizational silence. This technique effectively quantifies the strength and direction of linear relationships between continuous variables, allowing us to identify potential associations that inform further analyses. A SEM was conducted using AMOS Graphics, version 24.0, incorporating the bootstrap method with 5000 replicates to test the hypothesized model. SEM was selected because it can simultaneously assess multiple relationships among variables and test complex theoretical models, providing a comprehensive view of the interactions between organizational learning, organizational justice, and organizational silence.

In addition, multiple linear regression analysis was performed to evaluate the relationship between the dependent variable (organizational silence) and several independent variables, including demographic characteristics, organizational learning, and organizational justice. This technique was utilized to determine the extent to which these independent variables predict variations in the dependent variable. Three models were established as follows: Model 1 included demographic characteristics as independent variables to assess their impact on organizational silence. Model 2 focused on organizational learning as an independent variable. Model 3 examined organizational justice as another independent variable. The evaluation of the model's fit was based on established criteria to ensure the validity of the results. Specifically, we assessed the chi-square fit statistics divided by degrees of freedom (*χ*^2^/d*f*), aiming for a value less than 3, and the root mean square error of approximation (RMSEA), with a threshold of less than 0.08 [31]. Other fit indices included the goodness-of-fit index (GFI) > 0.90, comparative fit index (CFI) > 0.90, Tucker–Lewis index (TLI) > 0.90, normed fit index (NFI) > 0.90, and adjusted goodness-of-fit index (AGFI) > 0.80 [32]. Statistical significance was determined using a two-tailed test, with a *p* value of less than 0.05 considered significant.

### 3.5. Ethical Considerations

The study adhered to the ethical guidelines of the Declaration of Helsinki and was approved by the Ethics Committee of Ardabil University of Medical Sciences in Iran. All participants provided written informed consent, and the survey was conducted anonymously to safeguard their privacy.

## 4. Results

### 4.1. Participants' Characteristics

In this study, 319 clinical nurses took part. The average age of the participants was 30.96 ± 3.50 years. More than half were female (*n* = 174, 54.5%) and married (*n* = 180, 56.4%).  Table 2 provides a summary of the sociodemographic characteristics of the participants.

### 4.2. Correlation Analyses

The overall mean scores for organizational learning, organizational justice, and organizational silence among all participants were 100.37 ± 22.14, 38.39 ± 10.65, and 112.74 ± 25.84, respectively (Table 3).

A Pearson correlation coefficient was calculated to examine the relationship between organizational learning, organizational justice, and organizational silence. The results indicated a significant positive relationship between organizational learning and organizational justice (*r* = 0.907, *p* < 0.001). A significant negative correlation was found between organizational learning and silence (*r* = −0.760, *p* < 0.001). The findings also indicated a significant negative relationship between organizational justice and organizational silence (*r* = −0.793, *p* < 0.001). As a result, the research findings led to the confirmation of the research hypotheses. It is worth mentioning that the power of the evaluated relationships was above 0.6, indicating a strong relationship between organizational justice, organizational learning, and organizational silence. The correlation between organizational learning, organizational justice, and organizational silence is shown in the correlation matrix (Table 3).

### 4.3. Regression Analysis

Concerning organizational silence, in Model 1, the results showed that working experience, education level, and working department (emergency and ICU) were predictors of organizational silence in clinical nurses (Table 4). When the demographic variables were included in the model, the model *R*^2^ was equal to 0.618, suggesting that the demographic variables explained 61.8% of the variance in predicting organizational silence. In Model 2, all demographic variables and organizational learning were correlated with organizational silence. There was a significant improvement when the demographic variables and organizational learning were added to the model (*R*^2^ = 0.689, Δ*R*^2^ = 7.6%, Δ*F* = 38.213, *p* < 0.001). In the end, data in the final Model 3 showed that demographic variables (education level and working department), organizational learning, and organizational justice were significantly correlated with organizational silence. These independent variables explained 74.2% of the total variance of organizational silence (*R*^2^ = 0.742, Δ*R*^2^ = 4.8%, Δ*F* = 28.254, *p* < 0.001) (Table 4).

### 4.4. SEM

A SEM was constructed to determine the synthetic relationship between organizational learning, organizational silence, and organizational justice. Based on GFIs, the hypothesized conceptual model and its constituent concepts are acceptable overall: *χ*^2^ = 66.55, d*f* = 32, *p* < 0.001, *χ*^2^/d*f* = 2.080, RMSEA = 0.058, GFI = 0.960, CFI = 0.988, TLI = 0.984, NFI = 0.978, and AGFI = 0.931, and it showed that the model fit the data well (Table 5).

**Figure 2:**
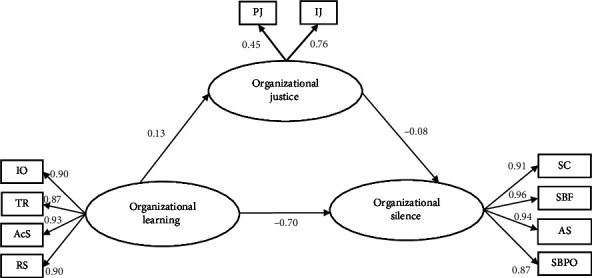
The structural equation modeling regarding the influence of organizational learning on organizational silence through organizational justice.

**Figure 3:**
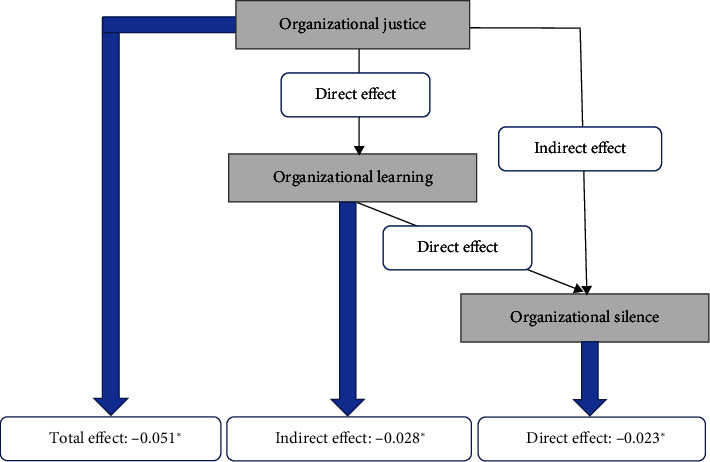
The total, indirect, and direct effects of organizational learning on organizational silence with organizational justice as a mediator (*n* = 319).

We used the bias-corrected bootstrap 95% confidence interval based on 5000 bootstrap samples to test the mediating effect. Organizational learning indirectly impacted (*β* = −0.028, *p* < 0.001) organizational silence mediated by organizational justice, with a 95% CI of −0.138 to −0.004. Since none of the 95% confidence intervals overlap with zero, it represents a significant difference. The direct effect of organizational learning on organizational silence was −0.023 (*p* < 0.001), so the mediating effect of organizational justice was partial. Therefore, since both the indirect path (organizational learning ⟶ organizational justice ⟶ organizational silence) and the direct path (organizational learning ⟶ organizational silence) became significant, we can conclude that only part of the impact of organizational learning on organizational silence was accounted for by organizational justice. The total effect of organizational learning on organizational silence was −0.051 (*p* < 0.001). The whole model explains 72.3% of all variance in organizational silence. The values of the total, indirect, and direct effects in the model are shown in Figure 2, Table 6, and Figure 3.

## 5. Discussion

Promoting open communication and active employee participation is essential to decrease organizational silence, which occurs when employees withhold their thoughts and concerns. Organizations can enhance their efficiency and productivity by creating an environment where employees feel valued and comfortable sharing their ideas. Our research focused on the relationship between organizational learning and silence in clinical nursing settings, specifically examining the influence of organizational justice. This study is the first to investigate the mediating role of organizational justice in the connections between organizational learning and nursing silence in a nursing context. This research contributes to the literature on reducing organizational silence.

Our research findings indicate that nurses demonstrated high levels of organizational learning, with average scores lower than those observed in Lyman's study [4]. One study suggested that the nurses involved perceived an average level of overall organizational learning [9]. Moreover, Miri et al. reported that nurses' organization learning is at an average level and above the average score [33]. Contrary to the current study's findings, Goula et al.'s research indicated minimal organizational learning in healthcare facilities [34]. The researchers believe this situation may be due to ongoing monthly training provided by training groups consisting of supervisors and hospital training instructors. The supervisors were keen to impart knowledge to the nurses to improve their performance. At the same time, the training instructors were more enthusiastic than the nurses in hospital centers regarding guidance and training. In addition, nurse managers actively coach their nurses to ensure the accurate performance of their duties.

According to the results of this study, the average score of organizational silence among nurses was at high levels, which is generally higher than other studies on nurses' organizational silence [1, 8, 9, 35]. Organizational silence refers to a phenomenon in which employees choose not to speak up or share their thoughts, ideas, or concerns due to a lack of psychological safety at work [36]. This result is to be expected because the lack of resources and high workload are signs that lead to organizational silence among nurses, which significantly contributes to increasing the organizational silence of employees [37]. The phenomenon of organizational silence within the context of Iranian nursing staff can be attributed to various factors, including the considerable pressures associated with their roles, the prevailing organizational culture, lack of resources, high workload, a lack of trust in management, and a shortage of safe spaces for open communication. Addressing this issue would involve the implementation of targeted educational programs aimed at fostering a culture of open dialog, as well as the creation of secure environments that encourage and facilitate discussions. These measures have the potential to significantly reduce organizational silence and consequently contribute to the enhancement of healthcare quality within this setting.

Our research findings showed that nurses rated organizational justice in medical education centers as average. Most research in the nursing workforce shows moderate organizational justice levels [38–40]. The observation can be attributed to various factors, including organizational culture, working conditions, and national culture. Creating a workplace culture that values and respects nurses' opinions and assessments and offers support to improve processes and working conditions can significantly increase nurse satisfaction and commitment. The implications of this can lead to a more comprehensive improvement in the quality of healthcare services overall.

This study explored the interconnection between organizational learning, organizational justice, and organizational silence. The study's results revealed a positive relationship between organizational learning and organizational justice, while a significant negative relationship exists between organizational learning and organizational silence. In addition, the study observed a significant negative correlation between organizational justice and silence. The research found that organizational learning has an indirect effect on organizational silence through organizational justice. This means that when an organization promotes learning, it also tends to foster a sense of fairness and equity, which in turn reduces the likelihood of employees remaining silent. It also has a direct impact on organizational silence. Although the mediating effect of organizational justice is only partial, it is still significant, indicating that it plays a crucial role in the relationship between organizational learning and silence. The researchers concluded that both the indirect path (organizational learning ⟶ organizational justice ⟶ organizational silence) and the direct path (organizational learning ⟶ organizational silence) are significant. However, since only part of the effect of organizational learning on organizational silence is accounted for by organizational justice, other factors may also contribute to the relationship.

### 5.1. The Direct Effect of Organizational Learning on Organizational Silence

There was a significant negative relationship between organizational learning and silence among nurses, consistent with Atalla, Elamir, and Abou Zeid's study [9]. Organizational silence is when nurses withhold information or avoid speaking out to protect their colleagues or organizations [8]. It is essential to create and maintain a positive work environment. However, it is crucial to speak up when patient safety is compromised. [41]. When an organization prioritizes continuous learning, it creates a culture of open communication that facilitates the transfer of knowledge and experiences [42]. This culture encourages people to speak up; share their opinions, ideas, and information; and avoid staying silent [43].

Moreover, when an organization welcomes different opinions and allows people to express themselves without fear of criticism or suggestions, it can help build trust and increase motivation to share information [44]. In general, organizational learning is critical in reducing organizational silence because it promotes sharing knowledge and experiences, thus improving the organization's ability to respond to new challenges and opportunities [45]. Encouraging continuous learning and open communication in the workplace is crucial for improving organizational performance and patient safety. Creating a work environment that welcomes diverse opinions and allows employees to express themselves without fear of criticism can help build trust and increase motivation to share information.

The findings confirmed that organizational justice was negatively associated with nurses' organizational silence, which aligns with previous studies [1]. Nurses are more likely to speak up about work-related issues when they feel their organization is fair and just [1]. In other words, promoting a work environment that values and prioritizes fairness and justice is critical in encouraging nurses to share important information, ideas, and feedback [46]. The study also discovered that nurses who view their organization as just and fair are less likely to engage in organizational silence. Organizational silence is when employees hold back valuable information or avoid speaking up about work-related issues due to concerns about job security, fear of retaliation, or a belief that their perspective will not be taken [47]. Developing a fair and just work environment is necessary to create a culture of openness, trust, and transparency among nurses. Fair compensation, recognition, career advancement, and a supportive, collaborative workplace culture can benefit nursing workforce engagement and empowerment. Encouraging nurses to share their perspectives can improve communication, problem-solving, and decision-making, ultimately enhancing patient care and outcomes.

Third, as this study hypothesized, organizational learning directly and positively impacted nurses' perceptions of organizational justice. This relationship has been observed for the first time. The study suggests that nurses who engage in continuous education and training are more likely to perceive greater fairness and justice in their workplace. This finding is important because it indicates that organizations prioritizing learning and development can foster a sense of justice among their employees. Organizational justice refers to how employees perceive the fairness of decisions and decision-making processes in the workplace and how they are treated by management [48]. Employees who feel that their organization treats them fairly and justly are more likely to be motivated, productive, and committed to achieving individual and organizational goals.

Moreover, this perception of justice can help reduce behavioral anomalies and improve overall organizational performance [49]. By encouraging employees to participate in organizational learning, managers can create a positive work environment where employees feel valued and supported [50]. However, since this result was observed for the first time in Iran, further studies are required to confirm and expand on these findings. Additional research can validate the relationship between organizational learning and organizational justice among nurses and provide a more comprehensive understanding of this relationship. Organizations should develop strategies to promote continuous learning and development, fostering a sense of justice and fairness in the workplace.

The fourth finding of the study indicates that organizational justice plays a positive and meaningful role in mediating the relationship between organizational learning and organizational silence. Organizational learning helps organizations acquire new knowledge, reconstruct current knowledge, and identify areas that require change [51]. It enables the organization to adapt, perform targeted activities, and make informed decisions [52]. Training and acquiring new knowledge make employees more productive and expressive and help avoid organizational silence, transforming the organization into a dynamic and efficient entity [53]. Organizational justice is crucial in promoting collective work spirit, enhancing employee motivation, and increasing human resource diligence [17]. Fair distribution of the organization's achievements improves employee morale and encourages them to put more effort into their activities [54]. Employees' perception of organizational justice is a fundamental factor that can influence their behavior and reduce organizational silence. However, further research is required to understand the relationship between these variables better and explain the results.

### 5.2. Limitations

Our research indicates that healthcare organizations can reduce silence by establishing an open communication culture and supporting a conducive work environment. The study affirms the significance of transparent communication in healthcare organizations and highlights the benefits of encouraging transparency and accountability. However, we acknowledge that our research has some limitations. The study relied on self-reported measures, which could introduce response bias and social desirability effects, possibly affecting the validity of the findings. In addition, the study's cross-sectional design limits our ability to establish causal relationships. Future research could benefit from longitudinal or experimental designs to explore the temporal dynamics of organizational learning, justice, and silence among nursing students. Comparative studies across cultural and organizational settings, complementing quantitative approaches with qualitative methods such as interviews or focus groups, could offer a more comprehensive understanding. Conducting intervention studies to evaluate the effectiveness of targeted educational programs, communication interventions, or organizational culture initiatives in promoting open communication among nursing students would be beneficial. Exploring the influence of additional variables such as leadership styles, organizational culture, and job satisfaction on organizational learning, justice, and silence could provide a more comprehensive understanding of nursing settings.

### 5.3. Implications to Nursing Management

Based on our results study, encouraging open communication and active employee participation is crucial for reducing organizational silence in healthcare organizations. Creating a work environment that values and prioritizes fairness and justice is critical in encouraging nurses to share important information, ideas, and feedback, ultimately improving patient care and outcomes. Promoting continuous learning and development can foster a sense of justice and fairness among nursing staff, leading to a positive work environment. Addressing factors such as high workload and a lack of resources is essential to reduce organizational silence among nurses and enhance healthcare quality. For better management practices, further research is needed to understand the complex interplay between organizational learning, justice, and silence in nursing settings.

## 6. Conclusion

Our study shows a positive connection between organizational learning and organizational justice and a negative link between learning and silence. Organizational justice was also found to have a negative relationship with nurses' silence. Despite showing high levels of organizational learning, nurses' average scores were lower than previous studies. In contrast, their average silence scores were higher due to factors such as heavy workload and lack of resources. These findings have significant social and economic implications. Creating a culture of open communication within healthcare organizations can help empower nurses to express their opinions and concerns, ultimately improving healthcare quality. It is recommended that hospital management focus on training programs and creating supportive environments to strengthen organizational learning and justice. Future research should investigate the impact of specific interventions on organizational learning and justice and include diverse perspectives from various healthcare professionals. Your unique insights are crucial to gaining a deeper understanding of these dynamics and improving healthcare practices.

## Figures and Tables

**Table 1:** Significant papers addressing the topics of the documents.

Authors	Objectives	Hypotheses	Findings
She et al. [1]	To explore the effects of ethical leadership on nurses' organizational silence and the mediating role of organizational justice	Ethical leadership positively influences organizational justice and negatively influences organizational silence	Ethical leadership significantly predicted nurses' organizational justice (*β* = 0.453, *p* < 0.001) and silence (*β* = −0.450, *p* < 0.001), with organizational justice partially mediating this relationship
Lyman and Ethington [4]	To examine developmental stages associated with organizational learning in hospitals	Organizational learning stages impact nurses' engagement and performance	Identified key developmental stages that enhance organizational learning, leading to improved performance outcomes in nursing
Atalla, Elamir, and Abou Zeid [9]	To explore the relationship between organizational silence and organizational learning in nurses	Organizational silence negatively impacts organizational learning among nurses	Higher levels of organizational silence correlated with decreased organizational learning capabilities
Gillet et al. [17]	To examine the mediating role of organizational justice in the relationship between transformational leadership and nurses' quality of work life	Organizational justice mediates the relationship between transformational leadership and quality of work life	Transformational leadership positively influenced organizational justice, which in turn enhanced nurses' quality of work life
Çaylak and Altuntas [14]	To analyze the impact of organizational silence among nurses on organizational cynicism and intention to leave work	Organizational silence is positively correlated with organizational cynicism and intention to leave	Higher levels of organizational silence were associated with increased organizational cynicism and a greater intention to leave
Hameed et al. [24]	To investigate the relationship between organizational justice and knowledge-sharing behavior among nurses	Organizational justice positively influences knowledge-sharing behavior, impacting organizational learning	Organizational justice significantly enhanced knowledge-sharing behavior, which is critical for effective organizational learning
Safari et al. [12]	To analyze relationships between organizational support, trust, and organizational commitment among nurses	Organizational support and trust positively influence organizational commitment and reduce silence	Higher levels of organizational support and trust were linked with increased organizational commitment and reduced silence behaviors

**Table 2:** Sociodemographic characteristics of the participants (*n* = 319).

Variables	Categories	Mean ± SD	
Age (years)		30.96 ± 3.50	

Working experience (years)		8.82 ± 3.24	

		No.	Percentage

Gender	Male	145	45.5
	Female	174	54.5

Marital status	Single	139	43.6
	Married	180	56.4

Education level	Bachelor's degree	230	72.1
	Master's degree	89	27.9

Working department	Medical	92	28.8
	Surgical	68	21.3
	Emergency	32	10.1
	ICU	34	10.7
	Pediatric	23	7.2
	Other	70	21.9

**Table 3:** Correlation matrix of organizational learning, organizational justice, and organizational silence (*n* = 319).

Variables	Mean	SD	1	2	3
			*r* (*p*)		
(1) Organizational learning	2.87	0.63	1		
(2) Organizational justice	2.95	0.81	0.907 (*p* < 0.001^∗^)	1	
(3) Organizational silence	3.52	0.80	−0.760 (*p* < 0.001^∗^)	−0.793 (*p* < 0.001^∗^)	1

*Note:* Numbers 1–3 in the title row represent the numbered variables in the first column.

⁣^∗^Correlation is significant at the 0.01 level (2-tailed).

**Table 4:** The hierarchical linear regression analysis coefficients to examine predictors of the organizational silence (*n *=* *319).

Variables	Model 1			Model 2			Model 3		
	*B*	Beta	*p*	*B*	Beta	*p*	*B*	Beta	*p*
Age	0.681	0.092	0.566	1.139	0.154	0.288	1.304	0.177	0.187
Working experience	−3.213	−0.403	0.012	−1.487	−0.187	0.199	−0.940	−0.118	0.380
Gender (male = 0^a^)	1.105	0.021	0.603	0.860	0.017	0.602	0.315	0.006	0.836
Marital status (single = 0^a^)	−0.398	−0.008	0.831	−0.463	−0.009	0.782	−0.850	−0.016	0.581
Education level (BSc = 0^a^)	−31.58	−0.549	0.001	−26.64	−0.463	0.001	−34.15	−0.594	0.001
Working department (medical = 0^a^)									
Surgical	2.115	0.034	0.375	−2.716	−0.043	0.219	−6.017	−0.096	0.004
Emergency	−29.25	−0.341	0.001	−10.38	−0.244	0.001	−23.24	−0.270	0.001
ICU	−30.67	−0.367	0.001	−11.54	−0.138	0.001	−24.28	−0.290	0.001
Pediatric	1.365	0.014	0.660	6.420	0.064	0.052	1.995	0.020	0.520
Other	−1.516	−0.022	0.507	1.881	0.027	0.434	−1.015	−0.015	0.655
Organizational learning				−0.911	−0.376	0.001	−0.077	−0.032	0.001
Organizational justice							−0.221	−0.190	0.001
Model characteristics	*R* = 0.786, *R*^2^ = 0.618, adjusted *R*^2^ = 0.609, *F* = 62.81, *p* < 0.001			*R* = 0.833, *R*^2^ = 0.694, Δ*R*^2^ = 0.076, adjusted *R*^2^ = 0.689, Δ*F* = 38.213, *p* < 0.001			*R* = 0.861, *R*^2^ = 0.742, Δ*R*^2^ = 0.048, adjusted *R*^2^ = 0.732, Δ*F* = 28.254, *p* < 0.001		

Abbreviations: B, unstandardized coefficient; Beta, standardized coefficient.

^a^Reference groups.

**Table 5:** Goodness-of-fit statistics for the structural equation model (*n* = 319).

Indices	*χ* ^2^	d*f*	*p-*value	*χ* ^2^/d*f*	RMSEA	GFI	CFI	TLI	NFI	AGFI
Values of model	66.55	32	0.001	2.080	0.058	0.960	0.988	0.984	0.978	0.931
Acceptable values	—	—	> 0.05	< 3	< 0.08	> 0.90	> 0.90	> 0.90	> 0.90	> 0.80

Abbreviations: *χ*^2^/d*f*, ratio of chi-square to its degree of freedom; AGFI, adjusted goodness-of-fit index; CFI, comparative fit index; GFI, goodness-of-fit index; NFI, normed fit index; RMSEA, root mean square error of approximation; TLI, Tucker–Lewis's index.

**Table 6:** Results for the total, indirect and direct effects of organizational learning on organizational silence with organizational justice as a mediator (*n* = 319).

Effect	Pathway	Estimated (*β*)	BC 95% CI	
			Lower	Upper
Total effect	c	−0.051∗	−0.268	−0.007
Indirect effect	ab	−0.028∗	−0.138	−0.004
Direct effect	cʹ	−0.023∗	−0.646	−0.006

Abbreviations: BC, bias-corrected; CI, confidence interval.

^∗^Significant at the 0.01 level (2-tailed).

## Data Availability

The data that support the findings of this study are available from the corresponding author (A.M.) upon request.
